# Fibrates and risk of congenital malformations: a nationwide cohort study in South Korea

**DOI:** 10.1007/s00404-023-07357-2

**Published:** 2024-03-29

**Authors:** Hee Yeon Kay, Ha Young Jang, In-Wha Kim, Jung Mi Oh

**Affiliations:** 1 College of Pharmacy and Research Institute of Pharmaceutical Sciences, Seoul National University, Seoul, Republic of Korea; 2 College of Pharmacy, Gachon University, Incheon, 21936 Republic of Korea

**Keywords:** Lipid-lowering agents, Drug-induced congenital abnormalities, Pregnancy complications, Retrospective cohort study, Hypertriglyceridemia

## Abstract

**Purpose:**

To examine the association between maternal prescriptions for fibrates and congenital malformations in live births.

**Methods:**

Nationwide retrospective cohort study was conducted using the data sourced from the Korean National Health Insurance database. A cohort of 756,877 completed pregnancies linked to live-born infants in 215,600 women with dyslipidemia between 2012 and 2021. The study compared data on congenital anomalies between pregnancies who were exposed to fibrates and those who were not exposed to fibrates in the first trimester. Odds ratios (OR) were calculated by a multivariable analyses using logistic regression models to adjust for potential confounders.

**Results:**

260 pregnancies (0.12%) were exposed to fibrates during the first trimester. The prevalence of malformations in exposed offspirng was 10.77%, not significantly different compared with 9.68% in offspring of women who were not prescribed fibrates during pregnancy in patients with dyslipidemia (OR 1.13; 95% CI 0.75–1.70).

**Conclusion:**

This study implies that the use of fibrates during pregnancy may be safe, as it did not show any association with congenital anomalies. However, caution is warranted due to an elevated risk associated with prolonged exposure.

**Supplementary Information:**

The online version contains supplementary material available at 10.1007/s00404-023-07357-2.

## What does this study adds to the clinical work?

**Table:** 

The study suggests that prolonged exposure to fibrates during pregnancy could potentially elevate the risk of congenital anomalies. Therefore, if deemed necessary, a shorter duration of prescription is recommended to minimize the risk. This research offers valuable insights for obstetricians in determining treatment approaches for pregnant women with dyslipidemia.	

## Introduction

Low-density lipoprotein cholesterol is a widely recognized causal factor in the development of atherosclerotic cardiovascular disease and represents the primary focus of lipid-lowering treatment [1]. There is, however, still a substantial risk of atherosclerotic cardiovascular disease (CVD) events despite intensive statin therapy, and the results from clinical trials suggest that an elevated concentration of triglycerides is a marker of residual cardiovascular risk on low-density lipoprotein-lowering therapy. Patients with nonfasting triglycerides concentrations > 443 mg/dL showed 3.4-fold increased risk of CVD (78 events/10,000 person years) compared to those with < 89 mg/dL [2]. Triglyceride levels are a significant risk factor for coronary artery disease in women, more so than in men [3].

Pregnancy-related hypertriglyceridemia is infrequent, but in certain cases, it can pose a life-threatening condition. A systematic review of prevalence of acute pancreatitis in pregnancy suggested that it occurs in 0.02–0.22% of pregnancies [4]. Complications can include acute pancreatitis, hyperviscosity syndrome, and possibly preeclampsia [5–7]. In diabetic pregnancy, exaggerated hypertriglyceridemia has been found compared to normal pregnancy, which has been correlated to neonatal body weight or fat mass [8]. Fibrates are practical first-line choice for patients with very high triglyceride levels (≥ 500 mg/dL), but only few case reports are available regarding patients been prescribed fibrates in pregnancy and their teratogenic outcomes [9–11]. The European Society of Cardiology/European Atherosclerosis Society guidelines currently contraindicate the use of fenofibrate in pregnant women due to a lack of substantial evidence [12]. While there's limited data on the usage of fibrates during pregnancy, their application remains a topic of debate because of potential teratogenic risks. No published reports have demonstrated teratogenic effects, and both fenofibrate and gemfibrozil are categorized as pregnancy category C drugs [5, 10, 13–15]. As of now, such categorizations are no longer widely utilized, and the FDA's stance is that fenofibrate should only be administered during pregnancy if the anticipated benefits outweigh the potential fetal risks [9]. Clinical trials targeting pregnant women are difficult since pregnant women are a vulnerable group. Furthermore, due to very low prevalence of exposure, the analysis of drug exposure cases during pregnancy requires nationwide data which made it difficult to conduct a cohort study.

Prevalence of hypertriglyceridemia increases with age, from 3.9% in women aged 20–29 to 12.6% in women aged 50–59 and the domestic prevalence rate is 15.0% in 2020 [16]. In a Taiwanese cohort study, 19% of pregnant women had fasting plasma TG levels exceeding 140 mg/dL in the first trimester, and the risk of developing adverse pregnancy outcomes (i.e. gestational diabetes mellitus or large for gestational age) was 2.56 times higher [17]. Safety data is required since the possibility of exposure in pregnant women is also increasing. The embryonic period, from the second to the eighth week of gestation, is the critical window of vulnerability for the developing fetus when exposure to harmful agents such as drugs, may cause structural malformations [18]. In pursuit of safe management of this patient group, we investigated the association between fibrates therapy during the first trimester and increased prevalence of congenital malformations.

## Methods

### Data source and study population

A cohort study was conducted on data from a nationwide customized health information data; all data were linked with the National Health Insurance Service (NHIS) [19] database which are collected, managed, and maintained by the NHIS to be modified as requested in the purpose of policy and academic research. The NHIS is the only and compulsory public medical insurance system operated by the Korean government. To claim payments for patients care, all clinics and hospitals in Korea submit data, including the patient's personal identification number, diagnosis, and prescription information, to the NHIS. The requirement for written informed consent from subjects was waived because all subjects were anonymized using a randomized identification number. This study was approved by the institutional review board of Seoul National University (IRB No. E2212/004–009). This study was done within the framework of the Korean Health Insurance Review and Assessment Service, which is responsible for claim reviews and quality assessment of the NHIS.

Our analyses included deliveries between January 2012 and December 2021 in women with dyslipidemia aged 19 to 44 years who did not have prior childbirth records for at least 1 year before the date of delivery. We defined dyslipidemia based on the presence of international classification of diseases 10th revision (ICD-10) diagnostic codes of E78.0-E78.9. The linked offspring records of women with delivered pregnancies (ICD-10 diagnostic codes of O80–O84) were also included, and cases that could not be linked in the NHIS were excluded. To rule-out the possible effect of clustering among siblings, only the firstborn infants of each mother were included. Pregnancies with exposure to known teratogenic drugs (e.g., systemic retinoids, antineoplastic agents, thalidomide, antiepileptic medications, misoprostol, warfarin) were excluded.

### Fibrates use

A prescription claim for fibrates within one year before the date of delivery was used for analyses. The start date of a pregnancy was predefined as 40 weeks (280 days) before the date of childbirth, which is the normal duration of a full-term human pregnancy, because the exact gestational age at birth was unknown. We operationally defined a child as exposed to maternal fibates in the first trimester if the mother had received at least 1 fibrate prescription during the first trimester (from 190 to 280 days before childbirth), known as the etiologically relevant period for congenital malformations. We considered the following fibrates: bezafibrate, gemfibrozil, and fenofibrate. Fibrates can be obtained only as prescription drugs in Korea. Fibrates and concomitant medications (e.g., antidiabetic agents, antihypertensives, antidepressants, statins) were defined based on ATC codes. The unexposed group comprised women who were not prescribed any fibrates from 3 months before the last menstrual period to the end of the first trimester.

### Outcomes

We included all hospitalizations and outpatient visits where congenital malformations were either the primary diagnosis or an additional diagnosis, as per the ICD 10 classification, with codes ranging Q00 to Q99. All congenital malformations were subcategorized in the following 11 major groups: Q00–Q07 (nervous system), Q10–Q18 (eye, ear, face, and neck), Q20–Q28 (circulatory system), Q30–Q34 (respiratory system), Q35–Q37 (cleft lip and cleft palate), Q38–Q45 (other digestive system), Q50–Q56 (genital organs), Q60–Q64 (urinary system), Q65–Q79 (musculoskeletal system), Q80–Q89 (other congenital malformations), Q90–Q99 (chromosomal abnormalities, not else classified).

### Statistical analysis

In the first trimester, women who used fibrates were matched with those who did not at a 1:10 ratio using a nearest neighbor greedy matching algorithm [20]. Propensity score adjustments were made for age, delivery year, maternal conditions (e.g., diabetes, hypertension), and concomitant medications (e.g., antidiabetic agents, antihypertensives, antidepressants, and statins, as detailed in Suppl Table 1). Standardized difference of over 0.1 was regarded as a sign of imbalance [21]. The prevalence difference of congenital malformations between the two groups was determined using the chi-square test. OR with 95% CIs were calculated using logistic regression to assess the risk of congenital malformations in infants who were exposed to fibrates compared to those in the comparison cohort. Logistic regression analyses were adjusted for BMI and smoking. First-trimester fibrates recipients were further categorized on the basis of drug duration and maternal age. Analyses were done using SAS version 9.4 (SAS Institute, Cary, NC, USA). A p-value less than 0.05 was considered statistically significant.

## Results

**Fig. 1:**
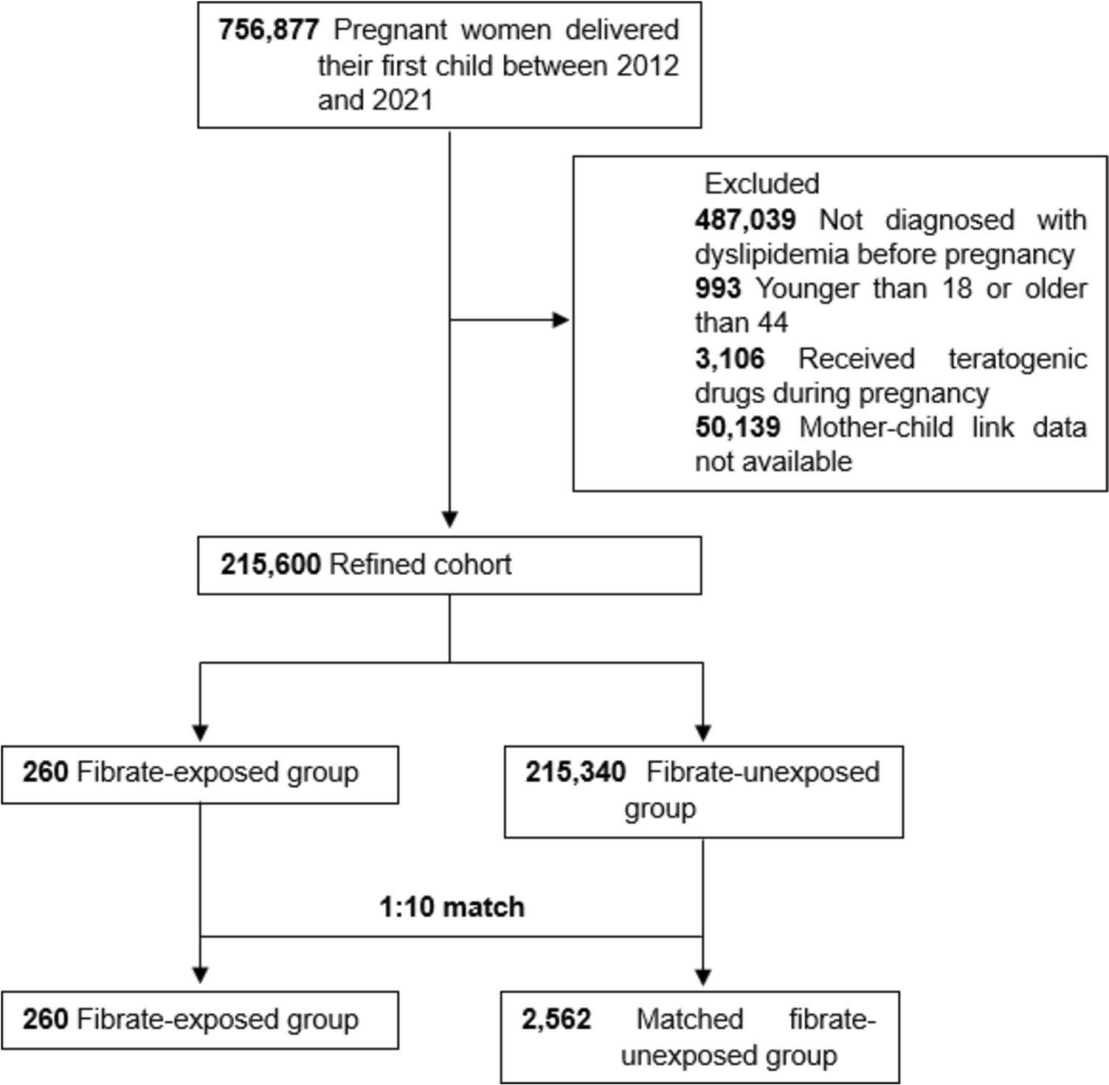
Study flow chart

Pregnancies from 2012 to 2021 in dyslipidemic women aged 19 to 44 years without childbirth records for the preceding year were included (*n* = 265,739), and 50,139 unlinked cases in the NHIS were excluded. The final study sample comprised 215,600 completed pregnancies linked to live-born infants (Fig. 1). Of those, 260 (0.12%) women received prescriptions for fibrates during the first trimester. Pregnancies in which women who had no prescription claims for fibrates during the first trimester were categorized as a comparison cohort (*n* = 215,340). Before matching, the fibrate-exposed group exhibited higher medical conditions and concomitant medication use. However, after matching, no significant baseline differences were observed, with standardized mean differences being less than 0.10 for all covariates (Table 1).

**Table 1:** Baseline characteristics of the cohort

Characteristics	Unadjusted			PS adjusted		
	Unexposed (*n*=215,340)	Fibrate-exposed (*n*=260)	SMD	Unexposed (*n*=2562)	Fibrate-exposed (*n*=260)	SMD
Mean age (SD), y	32.8 (4.4)	35.2 (4.1)	0.58	35.6 (4.1)	35.2 (4.1)	0.09
Birth year of infants, *n* (%)			0.55			0.05
2012	5,659 (2.6)	18 (6.9)		124 (4.8)	18 (6.9)	
2013	13,266 (6.2)	18 (6.9)		187 (7.3)	18 (6.9)	
2014	18,552 (8.6)	11 (4.2)		143 (5.6)	11 (4.2)	
2015	23,490 (10.9)	10 (3.9)		166 (6.5)	10 (3.9)	
2016	25,451 (11.8)	11 (4.2)		148 (5.8)	11 (4.2)	
2017	25,737 (12.0)	34 (13.1)		332 (13.0)	34 (13.1)	
2018	26,289 (12.2)	31 (11.9)		310 (12.1)	31 (11.9)	
2019	26,704 (12.4)	35 (13.5)		312 (12.2)	35 (13.5)	
2020	25,505 (11.8)	46 (17.7)		420 (16.4)	46 (17.7)	
2021	24,687 (11.5)	46 (17.7)		420 (16.4)	46 (17.7)	
Parity, n (%)			0.07			0.09
1	122,743 (57.0)	154 (59.2)		1630 (63.6)	154 (59.2)	
2	60,458 (28.1)	65 (25.0)		590 (23.0)	65 (25.0)	
≥3	32,139 (14.9)	41 (15.8)		342 (13.4)	41 (15.8)	
Maternal conditions, *n* (%)						
Diabetes	18,666 (8.7)	155 (59.6)	1.27	1538 (60.0)	155 (59.6)	0.01
Hypertension	7,355 (3.4)	84 (32.3)	0.81	825 (32.2)	84 (32.3)	0
Concomitent medications, *n* (%)						
Antidiabetic agents	4,871 (2.3)	132 (50.8)	1.32	1263 (49.3)	132 (50.8)	0.04
Antihypertensives	2,802 (1.3)	60 (23.1)	0.71	589 (23.0)	60 (23.1)	0
Antidepressants	2,855 (1.3)	13 (5.0)	0.21	131 (5.1)	13 (5.0)	0.01
Statins	2,509 (1.2)	73 (28.1)	0.82	699 (27.3)	73 (28.1)	0.02
Medical aid recipients, n (%)	716 (0.3)	2 (0.8)	0.06	20 (0.8)	2 (0.8)	0

*SD* standard deviation, *SMD* standardized mean differences

### Association with the congenital malformations

Overall, congenital malformations were detected in 28 (10.8%) pregnancies where fibrates were taken during the first trimester, compared with 248 (9.7%) in the unexposed group (crude OR 1.13; 95% CI 0.75–1.70) (Table 2). After adjusting for potential confounders, the OR estimates moved slightly toward a null value, indicating no discernible difference between the two groups (adjusted OR 1.06; 95% CI 0.70–1.60). Although it was a nationwide cohort study, the number of subjects were not enough to get sufficient statistical power for organ-specific analyses due to low exposure rate and low prevalence of each malformations. Instead, analyses were performed by separating malformations of circulatory system (i.e., the most frequently occurred) from the rest (Suppl Table 2). The risk for other congenital malformations except for circulatory system showed modest but non-significant increase in pregnancies exposed to fibrates during the first trimester (Table 2; OR 1.59; 95% CI 0.97–2.63).

**Table 2:** Prevalences of overall and congenital malformations of circulatory system and other organs in pregnancies exposed to fibrates during the first trimester

Variable	Unexposed (*n* = 2562), *n* (%)	Fibrate-exposed (*n*=260), *n* (%)	OR (95% CI)	aOR (95% CI)^a^
Overall congenital malformations (Q00–99)	248 (9.7)	28 (10.8)	1.13 (0.75–1.70)	1.06 (0.70–1.60)
Circulatory system (Q20–28)	228 (8.6)	25 (9.1)	1.06 (0.69–1.64)	0.96 (0.62–1.48)
Others (Q00–18, Q30–99)	118 (4.4)	19 (6.9)	1.59 (0.97–2.63)	1.56 (0.94–2.58)

*OR* Odds ratio, *CI* confidence interval, *aOR* adjusted odds ratio, *BMI* body mass index

^a^Adjusted for BMI, smoking

### Subgroup analysis

Analyses examining the duration of fibrates use or age-dependent malformations aligned with the primary findings. No risk difference in overall malformations was observed between groups. However, a difference was noted when fibrates were used for more than 60 days in early pregnancy (Table 3). Similar duration-response relation was observed for other malformations except for circulatory system. When excluding malformations of circulatory system, malformations increased duration- and age- dependently.

**Table 3:** ORs for congenital malformations in pregnancies exposed to fibrates during the first trimester, by duration and maternal age

Variable	Overall congenital malformations OR (95% CI)	Circulatory system (Q20–28) OR (95% CI)	Other (Q00–18, Q30–99) OR (95% CI)
Duration of fibrate exposure			
≤ 60 days	0.53 (0.26–1.10)	0.47 (0.22–1.02)	1.24 (0.59–2.60)
> 60 days	1.32 (1.03–1.69)	1.26 (0.97–1.63)	1.39 (1.01–1.92)
Maternal age at the time of pregnancy			
< 35 years	0.99 (0.49–1.98)	1.05 (0.52–2.11)	1.19 (0.49–2.89)
≥ 35 years	1.13 (0.67–1.89)	0.93 (0.53–1.63)	1.88 (1.02–3.49)

*OR* Odds ratio, *CI* confidence interval

### Sensitivity analysis

In sensitivity analyses that redefined exposure as two or more fibrate dispensings during the first trimester, our main findings remained consistent (Table 4). The risk of overall malformations, as well as subgroup analyses of circulatory system malformations and other organ malformations linked to early prenatal fibrate exposure, showed no significant difference compared to unexposed pregnancies.

**Table 4:** ORs for congenital malformations in pregnancies exposed to fibrates during the first trimester: a sensitivity analysis

Variables	Unexposed (*n* = 2,562), *n* (%)	Fibrate-exposed (*n* = 138), *n* (%)	OR (95% CI)	aOR (95% CI)^a^
Overall congenital malformations (Q00–99)	248 (9.7)	23 (16.7)	1.37 (1.08–1.73)	1.32 (1.04–1.67)
Circulatory system (Q20–28)	228 (8.6)	20 (13.6)	1.30 (1.01–1.66)	1.22 (0.95–1.57)
Others (Q00–18, Q30–99)	118 (4.4)	12 (8.2)	1.38 (1.02–1.89)	1.37 (1.00–1.87)

*OR* Odds ratio, *CI* confidence interval, *aOR* adjusted odds ratio, *BMI* body mass index

^a^Adjusted for BMI, smoking

## Discussion

In this study, pregnant women exposed to fenofibrate during the first trimester were followed up to assess the risk of congenital malformations. To the best of our knowledge, this is the first large cohort study using the NHIS’s mother–child linked data to confirm the fetal safety of fenofibrate. A total of 0.7 million pregnancies were reviewed, and our study found that fibrates exposure during the first-trimester, a critical window of vulnerability for congenital malformations, was not associated with a substantially increased risk of overall congenital malformations.

The observed null associations of overall malformations with early prenatal fibrate use were consistent with existing case reports. A case report showed that fenofibrate could be used to treat high triglyceride-associated pancreatitis in the last trimester of pregnancy [11]. In another case report, gemfibrozil was successfully used to ameliorate the patient‘s hypertriglycerides, with no adverse pregnancy complications [14]. As a result, if deemed necessary, fibrates can be administered cautiously to pregnant women. It is not known whether fibrates cross the human placenta, however, the molecular weight of the metabolite (~ 319 Da) and the prolonged elimination half-life suggest that the drug would cross the placental barrier [22]. Alternatively, the high serum protein binding might limit the amount available for transfer to the embryo or fetus.

In our study, the analysis of duration of fenofibrates use is noteworthy. Note that a longer duration of exposure (> 60 days) increased the risk of overall malformations. This same pattern was observed in malformations excluding those of the circulatory system. Fenofibrate has been shown to be embryocidal and teratogenic in rats when given in doses 7–10 times the maximum recommended human dose [23] and embryocidal in rabbits when given at 9 times the maximum recommended human dose, suggesting potential harmful effects of fenofibrate exposure during the first trimester on fetal development. Therefore, our research results do not necessarily mean that fenofibrate is absolutely safe. There’s a possibility that cumulative effects could appear with continuous use, so caution should be exercised.

According to the results revealed in our study, the overall malformation rate is 9.7% in the control group and 10.8% in the exposure group, which appears to be higher than the 5.5% reported by another study [24]. To determine the effect of patient characteristics of the cohort, prevalence of overall malformations were confirmed in the entire cohort of 215,600 patients. When analyzed in the entire cohort, teratogenicity was 4.9% suggesting high prevalence was due to underlying maternal condition of the study subjects (data not shown). In our analysis, fibrates appeared to be relatively safe in terms of effects on the circulatory system when compared to other potential areas of concern. One possible explanation for this observation might lie in the mechanism of action of fibrates. These drugs primarily act to reduce triglyceride levels and increase HDL cholesterol [25]. The impact of fibrates on the circulatory system, particularly during fetal development, might be mitigated by their specific pharmacodynamic properties. However, there may be aspects of the fibrate mechanism that haven’t been fully explored in relation to fetal development, especially concerning the circulatory system.

This study also has a few limitations, which should be carefully taken into account when interpreting our findings. First, we were unable to determine whether patients actually took the fibrates prescribed. Therefore, we applied a strict exposure definition of  ≥ 2 prescriptions of fibrates to address for this uncertainty. It was assumed that if a woman refilled the respective drug’s prescription, she probably took them. Second, while we used the entire population of South Korea, there werent enough cases to ensure statistical significance when analyzing all malformations by category. Further studies are needed regarding organ-specific malformations of fibrates in the future. Last, residual confounding by indication or from unmeasured confounders may still be present due to inherent limitations of the observational nature of this study using claims data.

Our analysis did not identify a significant teratogenic effect from the use of fibrates in the first trimester. This suggests that inadvertent use during this period might not be a major concern. However, exposure exceeding 60 days showed increased teratogenicity. More information on the long-term effects of in utero exposure to fibrates is essential. The impact on other neonatal outcomes also needs investigation. Moreover, replicating our findings in other large datasets—with detailed information on fibrate use, confounders, and outcomes—is crucial. Only with these further studies can fibrate use during pregnancy be deemed safe.

## Supplementary Information

Below is the link to the electronic supplementary material.

Supplementary file1 (DOCX 13 KB)

Supplementary file2 (DOCX 13 KB)

## Data Availability

The data that support the findings of this study are openly available at the online public repository of the National Health Insurance Sharing Service: https://nhiss.nhis.or.kr.

## Abbreviations

OR: Odds ratio
CVD: Cardiovascular disease
NHIS: National Health Insurance Service
ICD-10: International classification of diseases 10th revision
