# Supplementary material for: Fibrates and risk of congenital malformations: a nationwide cohort study in South Korea
Source: Arch Gynecol Obstet. 2024 Mar 29;310(4):1967–73. doi: 10.1007/s00404-023-07357-2 (PMC11393199; doi:10.1007/s00404-023-07357-2)
Supplement: Supplementary file 1 — Supplementary file1 (DOCX 13 KB) [file 404_2023_7357_MOESM1_ESM.docx]

**Suppl table 1. List of concomitant medications***

| **Medications** | **Unexposed (n=2,562),  n (%)** | **Fibrate-exposed (n=260), n (%)** |
| --- | --- | --- |
| **Antidiabetic agents**  Insulins and analogues  Biguanides (metformin)  Combinations of oral blood glucose lowering drugs (metformin+DPP-4 inhibitors) | 560 (21.9)  343 (13.4)  121 (4.7) | 53 (20.4)  23 (8.8)  12 (4.6) |
| **Antihypertensives**  Dihydropyridine derivatives (amlodipine)  Dihydropyridine derivatives (cilnidipine)  Angiotensin II receptor antagonists (losartan) | 119 (4.6)  85 (3.3)  69 (2.7) | 15 (5.8)  12 (4.6)  14 (5.4) |
| **Antidepressants**  Non-selective monoamine reuptake inhibitors (amitriptyline, nortriptyline)  Selective serotonin reuptake inhibitors (escitalopram)  Selective serotonin reuptake inhibitors (fluoxetine) | 281 (11.0)  153 (6.0)  84 (3.3) | 17 (6.5)  14 (5.4)  3 (1.2) |
| **Statins**  atorvastatin  rosuvastatin  pitavastatin | 286 (11.2)  203 (7.9)  37 (1.4) | 29 (11.2)  21 (8.1)  4 (1.5) |

* A list of major concomitant medications, ≥5% of exposure cases
