# Supplementary material for: Fibrates and risk of congenital malformations: a nationwide cohort study in South Korea
Source: Arch Gynecol Obstet. 2024 Mar 29;310(4):1967–73. doi: 10.1007/s00404-023-07357-2 (PMC11393199; doi:10.1007/s00404-023-07357-2)
Supplement: Supplementary file 2 — Supplementary file2 (DOCX 13 KB) [file 404_2023_7357_MOESM2_ESM.docx]

**Suppl table 2. Organ-specific congenital malformations in pregnancies exposed to fibrates during the first trimester**

| **Variable** | **Unexposed (n=2,562),  n (%)** | **Fibrate-exposed (n=260), n (%)** |
| --- | --- | --- |
| Nervous system (Q00-07) | 7 (0.3) | 0 (0) |
| Eye, ear, face and neck (Q10-18) | 9 (0.3) | 2 (0.7) |
| Circulatory system (Q20-28) | 228 (8.6) | 25 (9.1) |
| Respiratory system (Q30-34) | 1 (0.0) | 1 (0.4) |
| Cleft lip and cleft palate (Q35-37) | 3 (0.1) | 1 (0.4) |
| Digestive system (Q38-45) | 29 (1.1) | 5 (1.8) |
| Genital organs (Q50-56) | 8 (0.3) | 0 (0) |
| Urinary system (Q60-64) | 19 (0.7) | 1 (0.4) |
| Musculosksletal system (Q65-79) | 30 (1.1) | 5 (1.8) |
| Other congenital malformations (Q80-89) | 6 (0.2) | 1 (0.4) |
| Chromosomal abnormalities (Q90-99) | 6 (0.2) | 6 (1.1) |
